# Effect of High Hydrostatic Pressure (HHP) on the Enzymatic Hydrolysis of Fish Gelatin

**DOI:** 10.1002/biof.70042

**Published:** 2025-08-01

**Authors:** Ilhami Okur, Mecit Halil Oztop, Hami Alpas

**Affiliations:** ^1^ Department of Food Engineering Middle East Technical University Ankara Turkey; ^2^ Department of Food Science and Technology University of Nebraska‐Lincoln Lincoln Nebraska USA

**Keywords:** antioxidant capacity, enzymatic hydrolysis, fish gelatin, FTIR, high hydrostatic pressure

## Abstract

Fish gelatin offers an alternative source for gelatin production; however, it possesses weaker functional properties, such as a low melting temperature and gel strength. Protein hydrolysates, produced through the hydrolysis of food proteins, demonstrate a wide range of biological activities, including antihypertensive, hypocholesterolemic, antimicrobial, and antioxidant properties. High hydrostatic pressure (HHP) is a non‐thermal technology that can modify protein structures by inducing unfolding, thereby enhancing enzymatic accessibility and hydrolysis efficiency. Therefore, this study aimed to investigate the effects of different HHP parameters (400 and 500 MPa for 5, 15, and 30 min) and different Alcalase concentrations (2 and 4% wt/vol) on the production of fish protein hydrolysate. The results showed that HHP‐assisted enzymatic hydrolysis increased the degree of hydrolysis and antioxidant capacity. However, when the pressure increased from 400 to 500 MPa, the degree of hydrolysis and antioxidant capacity decreased. FTIR spectroscopy was used to characterize the secondary structural changes of gelatin during HHP‐assisted hydrolysis. The spectra revealed that more visible peaks of fish gelatin hydrolysate samples between 1000 and 1100 cm^−1^ attributed to the asymmetric stretching of phosphate group (PO_4_^3−^) were observed. Protein unfolding is an important factor in increasing HHP‐assisted hydrolysis. In conclusion, HHP combined with enzymatic hydrolysis is a promising alternative to produce protein hydrolysates with improved properties.

## Graphical abstract

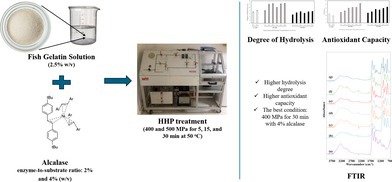

## 1 Introduction

More than 50% of the biomass generated from fish processing is discarded as waste annually. This substantial market, which involves the production of 140 million tons of fish, poses a significant environmental challenge. This by‐product can be processed into gelatin [1, 2]. Gelatin has the most significant value in the food industry, serving as a thickener, stabilizer, emulsifier, and gelling agent. It is a natural macromolecule produced from the collagen found in animal skin, bone, or white connective tissue through heating, partial hydrolysis, and denaturation. The commercial gelatin production is primarily from the skin or bone of porcine and bovine. Due to religious preferences that discourage the use of porcine and bovine gelatins among many populations worldwide, there has been a growing trend toward using fish skin and bone materials as alternative sources for gelatin production in recent years [3, 4]. Gelatins consisting of lower molecular weight peptides, such as gelatin hydrolysates, have been demonstrated to exhibit enhanced biological activities. Additionally, gelatin hydrolysates are highly valued in the food and pharmaceutical industries for their health benefits, including antioxidant properties and the ability to mitigate hypertension. Consequently, there is significant interest in utilizing gelatin hydrolysates as functional food additives [5, 6, 7].

There are several methods, such as chemical hydrolysis, enzymatic hydrolysis, and microbial fermentation, to produce hydrolysate [8, 9, 10]. Chemical hydrolysis, such as acylation, glycosylation, and covalent cross‐linking, can significantly enhance the functional properties of proteins by altering their structural configuration. However, the presence of off‐putting odors from hydrolysates and residual chemical reagents may diminish the commercial viability of these modified proteins [11, 12]. Like chemical hydrolysis, microbial fermentation has some disadvantages. Factors such as sugar type in the environment, presence of nutrition and oxygen, time, and the occurrence of competitive microorganisms are important factors for microbial fermentation. Therefore, suitable parameters have to be addressed before microbial fermentation. Moreover, microorganism enzymes are important for releasing bioactive peptides. Therefore, microbial fermentation is challenging due to poor reproducibility between batches compared to enzymatic hydrolysis [13, 14, 15]. Therefore, enzymatic hydrolysis seems to be the most effective modification method among these methods, especially for the large scale [16, 17].

To increase the efficiency of enzymatic hydrolysis, high hydrostatic pressure (HHP) has become a research focus [12, 17, 18, 19, 20, 21]. HHP is a non‐thermal food processing technique designed to enhance food safety and extend the shelf life while preserving freshness and nutritional value [22, 23]. This method is one of the most commercialized nonthermal techniques in the food industry, using high pressure, up to 1000 MPa, with a pressure‐transmitting medium‐filled vessel to package foods for a specified duration and temperature [24, 25, 26]. Protein denaturation due to HHP is a complex mechanism influenced by different factors such as protein structure, level of pressure, temperature, pH, and solvent polarity, wherein electrostatic and hydrophobic interactions within protein molecules may be altered [17]. The application of high pressure induces the deprotonation of charged groups and disrupts salt bridges and hydrophobic interactions, leading to conformational and structural changes in proteins. This results in aggregation and gelation [4, 27]. The integration of HHP with hydrolysis using enzymes in the production of protein hydrolysates decreases both the required enzyme quantities and reaction time, thereby enhancing hydrolysate yields since HHP promotes structural changes in the substrate, which can facilitate enzyme access [28, 29, 30, 31].

In the literature, there is limited information about the effect of combined enzymatic hydrolysis and HHP treatment on fish gelatin hydrolysis, especially about higher‐pressure levels (400 and 500 MPa). Thus, the scope of the current study was to evaluate the effect of different pressure levels, time, and enzyme concentration in the HHP‐assisted enzymatic hydrolysis using alcalase on fish gelatin. The fish gelatin hydrolysates were then tested for antioxidant activities. Also, the hydrolysate obtained with HHP‐assisted hydrolysis was characterized in terms of secondary structure by Fourier transform–infrared (FTIR) spectroscopy.

## 2 Materials and Methods

### 2.1 Materials

Fish gelatin (Custom Collagen, USA) is purchased from a local market in Turkey. Alcalase (declared enzyme activity ≥ 0.75 AU/mL) was obtained from Sigma‐Aldrich (St. Louis, MO, USA). All other reagents and solvents were obtained from Sigma‐Aldrich Company (St. Louis, MO, USA) and were analytical or chromatographic grade.

### 2.2 Fish Gelatin Hydrolysis

Fish gelatin hydrolysates were prepared according to Alemán et al. [1] with some modifications. Briefly, fish gelatin solutions (2.5% wt/vol) were prepared by mixing gelatin with sodium phosphate buffer solution, 0.1 M, pH 8. Fish gelatin was hydrolyzed with alcalase with two different enzyme‐to‐substrate ratios: 2% and 4% (wt/vol). Before hydrolysis, the mixture was preheated to the desired temperature, and pH was adjusted (50°C, pH 8). Then the alcalase enzyme was added to the solution. The hydrolysis reaction for control samples was done using a shaking water bath at 120 rpm and 50°C. At the end of enzymatic hydrolysis, the mixture was kept in hot water (90°C) for 15 min to inactivate the enzyme. After 15 min, samples were centrifuged at 8000 *g* for 20 min at 10°C to separate inactivated enzymes. Samples were kept at 4°C prior to analysis. Samples enzymatically hydrolyzed using the method above were indicated as a control.

For enzymatic hydrolysis under HHP, 760.0118 type pressure equipment supplied by SITEC‐Sieber Engineering, Zurich, Switzerland, was used. The instrument is equipped with a vessel (100 mL) with a 24 mm inner diameter and 153 mm length. The vessel temperature was measured by a thermocouple type K to maintain and control the processing temperature using a built‐in heating–cooling system (Huber Circulation Thermostat, Offenburg, Germany). Distilled water was used as a pressure‐transmitting medium. The pressurization rate was 340 MPa/min for 400 MPa, and the pressure release time was less than 20s for each condition. The pressurization and the pressure release times were not counted while reporting the processing time. Completely dissolved fish gelatin solution (2.5% wt/vol) in sodium phosphate buffer solution (0.1 M, pH 8) was poured into 25 mL sterile polyethylene cryotubes (LP Italiana SPA) and alcalase enzyme (2, and 4%) was mixed before pressurization. The enzymatic hydrolysis under high pressure was conducted at two different pressure levels (400 and 500 MPa) for three different processing times (5, 15, and 30 min) at a constant temperature (50°C). Samples after HPP were put into a 90°C shaking water bath to inactivate the enzyme for 15 min. After inactivation, samples were centrifuged for 20 min at 8000 *g* to separate inactivated enzymes. Samples were kept at 4°C until further analysis.

### 2.3 Determination of Degree of Hydrolysis by TNBS Colorimetry

The fish gelatin hydrolysate samples were prepared as mentioned above. The hydrolysis degree of the fish gelatin hydrolysate samples prepared using HHP and the traditional method was analyzed using a colorimetric method using TNBS [32]. 2.0 mL of TNBS reagent consisting of 0.1% (wt/vol) TNBS in water was mixed with 0.25 mL of fish hydrolysate sample and 2.0 mL of sodium phosphate buffer (0.2125 M, pH 8.2) in a test tube. Each tube was covered to protect against light and kept in a water bath for incubation at 50°C for 60 min. After completing 60 min incubation time, 4 mL of HCl (0.1 N) solution was added to stop the reaction. The samples were cooled down to room temperature before measuring absorbance. After cooling time, the absorbance value of the samples was measured using a spectrophotometer (Shimadzu UV‐1700, Japan) at 420 nm.

The standard curve was plotted using L‐leucine (0–2.0 mM). Degree of hydrolysis (DH) values were calculated using the equation below.

(1) $$\mathrm{DH}\left(\%\right)=\left(\frac{{\text{AN}}_{2}-{\text{AN}}_{1}}{N_{\mathrm{pb}}}\right)\times 100$$

where AN_1_, the amount of amino nitrogen of the sample before hydrolysis (mg g^−1^ protein). AN_2_, the amount of amino nitrogen of the sample after hydrolysis (mg g^−1^ protein). N_pb_, the nitrogen content of the peptide bonds in the protein substrate (mg g^−1^ protein). A value of 155.5 was used for fish gelatin [32].

### 2.4 Total Antioxidant Capacity

The total antioxidant capacity of the fish gelatin hydrolysate samples was measured using a DPPH (1,1‐diphenyl‐2‐picrylhydrazyl) solution based on Tkaczewska et al. [33]. Each fish gelatin hydrolysate sample (1.8 mL) was mixed with 1.8 mL DPPH (0.15 mM) in 95% ethanol. After mixing, samples were kept in a dark place for 30 min at room temperature for incubation. After a 30 min waiting time, the absorbance of the samples was measured at 517 nm using a spectrophotometer (Shimadzu UV‐1700, Japan). The total antioxidant capacity of the samples was calculated using the equation below.

(2) $$\%\text{inhibition of DPPH activity}=\left[1–\left(A_{s}/A_{c}\right)\right]\;x\;100$$

where A_s_, the absorbance value of the sample. A_c_, the absorbance value of the control.

### 2.5 Fourier‐Transport Infrared Spectroscopy (FTIR) Analysis

FTIR analyses were performed using dry samples. Samples were dried using a freeze dryer (Zhejang Value Mechanical & Electrical Products Co. Ltd., Wenling City, China) for 2 days before analyses. After lyophilization, samples were analyzed using an IR Affinity‐1 Spectrometer attached with attenuated total reflectance (ATR) (Shimadzu Corporation, Kyoto, Japan) between the 400 and 4000 cm^−1^ range with 4 cm^−1^ resolution and 32 scans.

### 2.6 Statistical Analysis

Minitab software package (SigmaPlot ver.14, Chicago, IL, USA) was used for all analyses. Results were analyzed using analysis of variance (ANOVA) and expressed as mean ± standard deviation. Means were compared with Tukey's multiple range test to find the significant differences between the levels of the same factors (*p* ≤ 0.05). Also, the Pearson correlation (α ≤ 0.05) was used to find the correlation coefficient between the degree of hydrolysis and antioxidant capacity.

## 3 Results and Discussion

### 3.1 Degree of Hydrolysis (DH)

**FIGURE 1:**
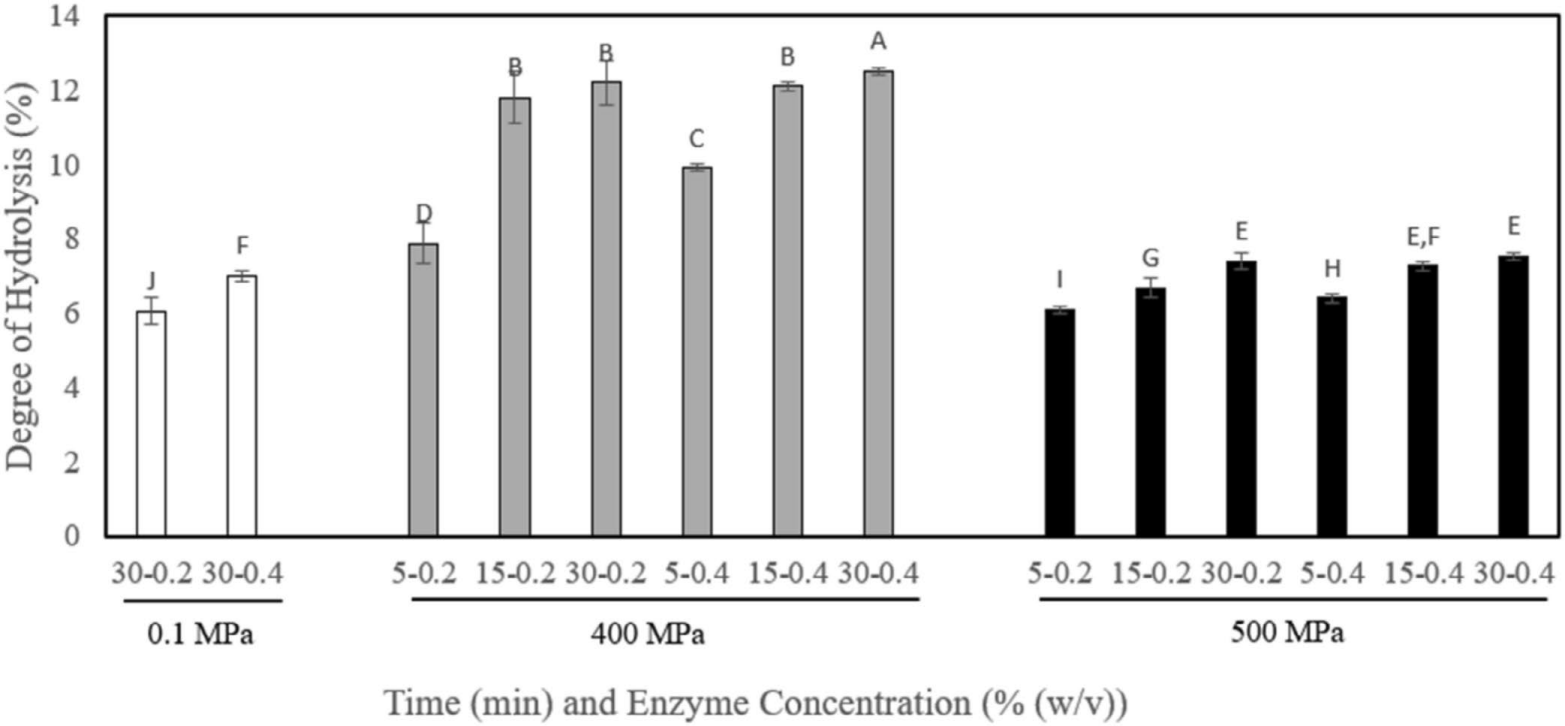
Hydrolysis degree (HD (%)) of fish gelatin hydrolyzed with alcalase at ambient pressure (untreated samples) and obtained with high hydrostatic pressure treatments at 400 and 500 MPa for 5, 15, and 30 min.

The DH results were shown in Figure 1. Regarding the results, HHP assisted enzymatic hydrolysis increased the DH of fish gelatin significantly compared to the control (*p* ≤ 0.05). The highest DH, 12.5%, was observed in 400 MPa for a 30 min treatment combination at 4% enzyme concentration. Also, the pressure level and holding time had a significant effect on the DH (*p* ≤ 0.05). On the other hand, enzyme concentration did not have a significant effect on DH (*p* = 0.076). Unlike thermal treatments, which affect covalent and non‐covalent bonds, HHP primarily affects weaker chemical bonds, such as hydrogen, hydrophobic, and ionic bonds. As a result, HHP causes native proteins denaturation and influences the interaction of protein–protein and protein‐solvent [34, 35]. Specifically, pressurization leads to the formation of monomeric, oligomeric, and aggregated species without using chemicals or elevated temperatures. Since covalent bonds, such as disulfide and peptide bonds, remain unaffected by HHP, the primary structure of proteins is preserved. Low‐pressure treatment (< 400 MPa) increases the number of hydrogen bonds, while higher pressures (> 400 MPa) disrupt them. Consequently, HHP selectively influences the secondary structure of the protein, with modifications of structure being reversible or irreversible depending on the process conditions [20]. These changes in protein structure can facilitate enzyme access, and this raises the hydrolysis rate. In the literature, many studies show that hydrolysis assisted by HHP increases the hydrolysis rate [17, 19]. HHP‐assisted enzymatic hydrolysis using Alcalase increased the DH up to 62.9% to produce quinoa protein hydrolysates with pressure levels between 200 and 600 MPa at 50°C for 15 min. De Maria et al. [36] studied the effects of HHP processes (100–500 MPa for 5–25 min) applied to bovine serum albumin while undergoing hydrolysis with α‐chymotrypsin. It was observed that the highest DH value was found as 10% at 400 MPa for 25 min, while the highest enzymatic hydrolysis was found as around 1%.

Pressure intensities of 500 MPa exhibited a negative impact on hydrolysis performance compared to 400 MPa treatment. This is likely attributed to the formation of soluble protein–protein aggregates. A greater degree of protein unfolding may occur at higher pressure levels, such as 500 MPa, followed by molecular rearrangement. This rearrangement can result in the recovery of the native structure or the formation of aggregates through exposed hydrophobic groups during or after processing [36, 37, 38, 39]. These aggregates could decrease the accessibility of the enzymes to the peptide bonds, so DH at 500 MPa pressure level was lower than the pressure level of 400 MPa.

Increasing treatment time caused a higher hydrolysis rate. Liu et al. [12] showed DH increased from 10.3% to 17.4% when HHP treatment time increased from 5 to 30 min for rice proteins with a pressure level of 300 MPa. Ambrosi et al. [17] found similar results and indicated that the highest DH value was observed at 30 min treatment time with the 400 MPa pressure treatment for whey protein. De Maria et al. [36] also found that the higher HHP treatment time leads to higher DH with the time interval of 5–25 min by varying pressure levels from 100 to 500 MPa.

### 3.2 Total Antioxidant Capacity

**FIGURE 2:**
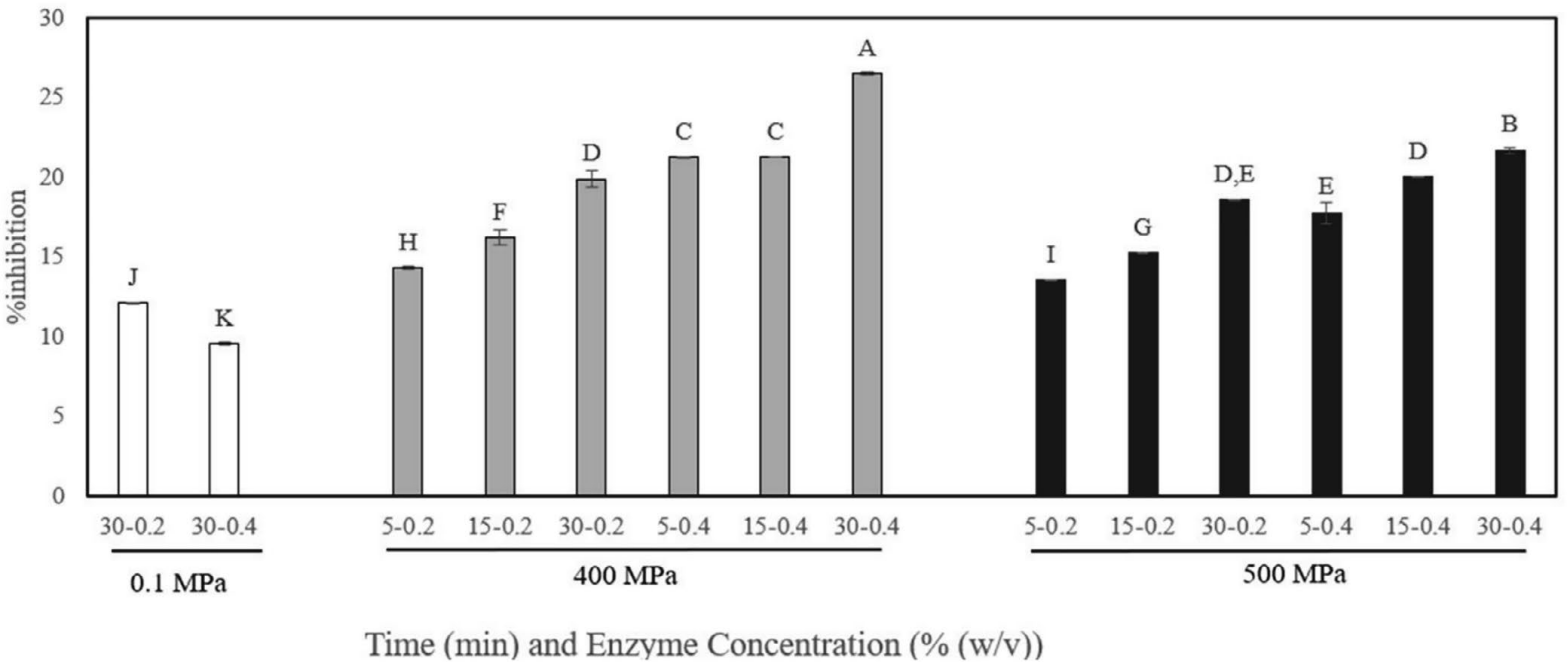
Antioxidant capacity of fish gelatin hydrolyzed with alcalase at ambient pressure (untreated samples) and obtained with high hydrostatic pressure treatments at 400 and 500 MPa for 5, 15, and 30 min.

Antioxidant capacity results of different hydrolysis techniques were reported in Figure 2. According to the results, HHP‐assisted enzymatic hydrolysis increased the antioxidant capacity of fish gelatin significantly compared to the control (*p* ≤ 0.05). Also, the pressure level and holding time had a significant effect on the antioxidant capacity (*p* ≤ 0.05). Like DH, the highest antioxidant capacity was found in 400 MPa for 30 min with 4% alcalase treatment (Figure 2). Furthermore, it was found that there was a correlation between antioxidant capacity and DH (*r* = 0.553). In the context of proteases, Alcalase is classified as a “serine endopeptidase,” which refers to its catalytic structure characterized by the classical catalytic triad of amino acids. This enzyme cleaves proteins in the middle of the amino acid chain [40, 41]. Alcalase is frequently utilized to generate peptides with predominantly hydrophobic characteristics [42]. 1,1‐Diphenyl‐2‐picryl Hydrazyl (DPPH) is considered a hydrophobic free radical. Thus, the presence of more hydrophobic peptides in the hydrolysate is expected to enhance DPPH scavenging activity [30, 43]. Therefore, it can be said that more hydrophobic peptides were hydrolyzed during HHP‐assisted enzymatic hydrolysis. Similar results were found in the literature for quinoa protein hydrolysates, sweet potato protein hydrolysates, and flaxseed protein isolate [19, 44, 45]. The hydrophobic peptides because of HHP‐assisted enzymatic hydrolysis also explain why there was a correlation between antioxidant capacity and DH.

### 3.3 Fourier‐Transport Infrared Spectroscopy (FTIR) Analysis

**FIGURE 3:**
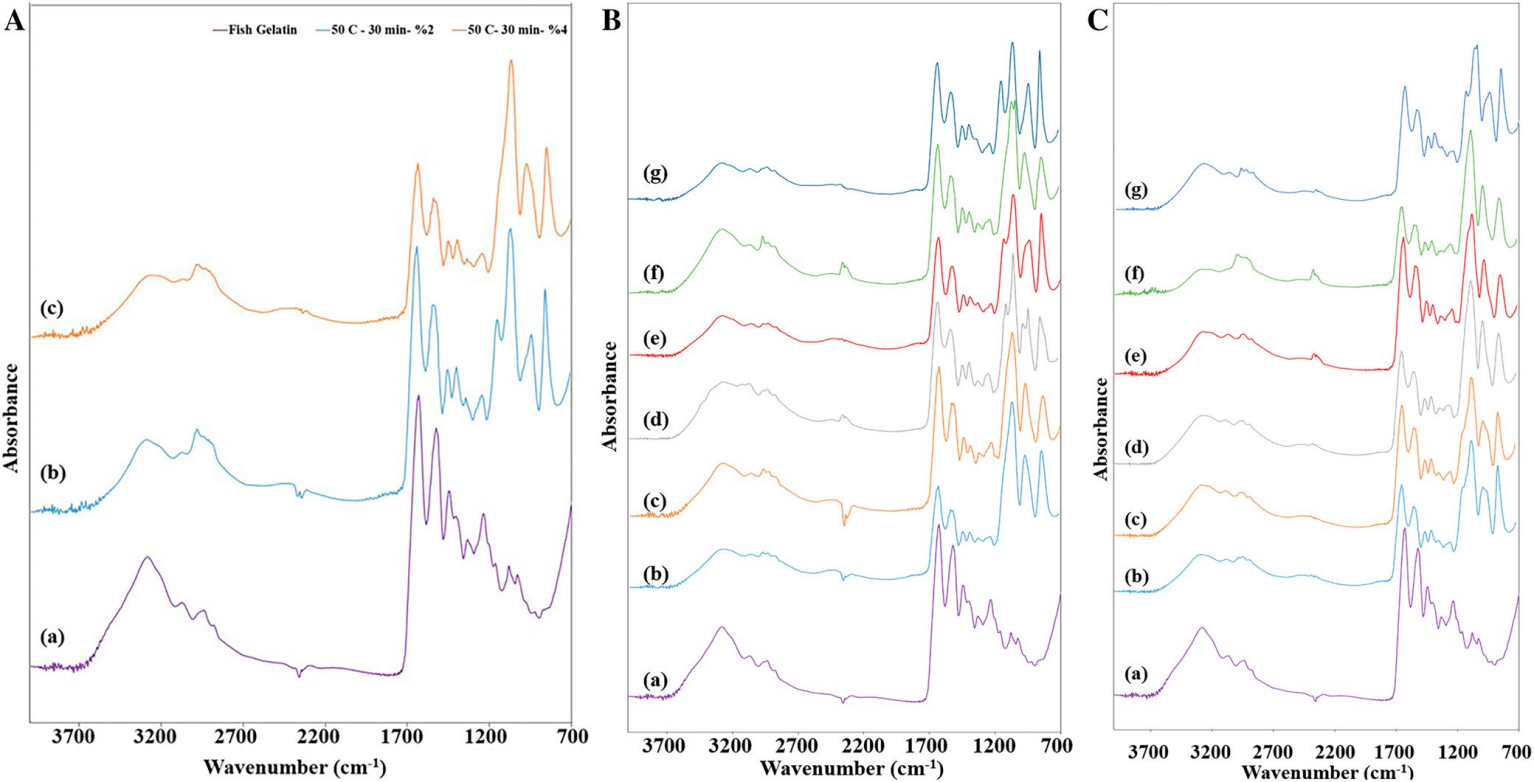
(A) FTIR spectra of fish gelatin hydrolyzed with alcalase at ambient pressure. (B) FTIR spectra of (a) Fish Gelatin, (b) 400 MPa—5 min—2% enzyme concentration, (c) 400 MPa—15 min—2% enzyme concentration, (d) 400 MPa—30 min—2% enzyme concentration, (e) 500 MPa—5 min—2% enzyme concentration, (f) 500 MPa—15 min—2% enzyme concentration, (g) 500 MPa—30 min—2% enzyme concentration. (C) FTIR spectra of (a) Fish Gelatin, (b) 400 MPa—5 min—4% enzyme concentration, (c) 400 MPa—15 min—4% enzyme concentration, (d) 400 MPa—30 min—4% enzyme concentration, (e) 500 MPa—5 min—4% enzyme concentration, (f) 500 MPa—15 min—4% enzyme concentration, (g) 500 MPa—30 min—4% enzyme concentration.

FTIR spectroscopy analyses were performed using fish gelatin and fish gelatin hydrolysates obtained after different hydrolysis conditions (Figure 3A,B), namely, pressure (400 and 500 MPa), time (15 and 30 min), and enzyme concentration (2% and 4%). The FTIR spectra of the fish gelatin sample revealed the typical band regions as amide A, amide I, II, and III bonds. Fish gelatin had an amide A band at a wavenumber of 3270.42 cm^−1^. The bands that appeared at 3270 cm^−1^ mainly corresponded with the free O—H groups and amine stretching [46]. The spectrum of samples showed that amide A, B (2933–2940 cm^−1^), and —CH_2_ stretch (2853 cm^−1^) tended to join. Previously, Shiao et al. [5] reported that this tendency may be due to dimeric intermolecular associations of carboxylic groups. This behavior was shown for all the samples regardless of the hydrolysis process. The rest of the bands of fish gelatin as amide I, II, and III were located at 1630.91, 1525.05, and 1241.78 cm^−1^, respectively. Sezer et al. [4] also reported similar FTIR spectra for fish gelatin with amide A, I, II, and III. The amide I, II, and III bands corresponded to C=O stretching, N—H bending, and C—N stretching, respectively [47]. The amide I band corresponds to the secondary structure of proteins as α‐helix, β‐sheet, and β‐turn, while the amide III band was about triple helix structure [48]. The hydrolysis of fish gelatin did not change the wavenumber of amide I, II, and III. The difference in the spectrum of those regions related to differences in chain conformation which did not occur after the hydrolysis of fish gelatin [49].

On the contrary, fish gelatin hydrolysate samples obtained using HHP or conventional methods exhibited more visible peaks between 1100 and 850 cm^−1^. More visible peaks of fish gelatin hydrolysate samples between 1000 and 1100 cm^−1^ attributed to the asymmetric stretching of the phosphate group (PO_4_^3−^) [50]. Previously, Zhang et al. [51] investigated the recovery of protein from the tilapia scale by using hydrothermal pretreatment and enzymatic hydrolysis. FTIR spectra of hydrothermal pretreated products obtained in the study showed the same peaks related to PO_4_^3−^ group.

## 4 Conclusions

In this study, the combined effects of limited enzymolysis and HHP treatment on the hydrolysis of fish gelatin were investigated. Through alcalase‐catalyzed hydrolysis and HHP treatment, both the degree of hydrolysis and antioxidant capacity were improved significantly (*p* ≤ 0.05). The best treatment condition was selected as 400 MPa for 30 min with 4% alcalase, where the highest degree of hydrolysis and antioxidant capacity were observed. Based on the FTIR results, a more visible peak related to the phosphate group (PO_4_^3−^) was observed. In brief, HHP‐assisted enzymatic hydrolysis is an effective approach to enhance the degree of hydrolysis and antioxidant properties of fish gelatin. However, further research is important to optimize the process parameters. Also, a techno‐economic analysis is required to understand the potential of this technology for scaling up.

## Data Availability

The data that support the findings of this study are available from the corresponding author upon reasonable request.
